# Comparison of Three Methods to Determine the Degree of Substitution of Quinoa and Rice Starch Acetates, Propionates, and Butyrates: Direct Stoichiometry, FTIR, and ^1^H-NMR

**DOI:** 10.3390/foods9010083

**Published:** 2020-01-12

**Authors:** Nabilah Abdul Hadi, Berthold Wiege, Sebastian Stabenau, Ali Marefati, Marilyn Rayner

**Affiliations:** 1 Department of Food Technology, Engineering, and Nutrition, Lund University, SE 22100 Lund, Sweden; ali.marefati@food.lth.se (A.M.); marilyn.rayner@food.lth.se (M.R.); 2 School of Food Science and Technology, University Malaysia Terengganu, Kuala Terengganu 21030, Terengganu, Malaysia; 3 Department of Safety and Cereals, Max Rubner-Institut, Federal Research Institute of Nutrition and Food, 32756 Detmold, Germany; Berthold.Wiege@mri.bund.de (B.W.); S.Stabenau@web.de (S.S.)

**Keywords:** modified starch, short-chain fatty acid, degree of substitution, stoichiometry, FTIR, ^1^H-NMR

## Abstract

Rice and quinoa starch esters were prepared by acylation using short-chain fatty acid anhydrides with different chain lengths (acetic, propionic, and butyric anhydride). A direct stoichiometric method based on the acylation reaction was used to determine the degree of substitution (DS) and acyl content (AC). In addition, Fourier-transform infrared spectroscopy (FTIR) was used to validate the conformational changes of acylated starch and ^1^H-NMR was used as a DS reference method. DS by stoichiometric calculation was shown to be in agreement with FTIR and was comparable with DS obtained from Proton nuclear magnetic resonance (^1^H-NMR). Based on this study, stoichiometric calculation allows rapid and direct determination of substitution levels and acyl content without the loss of samples, which provides efficiency and optimization of manufacturing procedures in producing the desired level of esterified starches.

## 1. Introduction

Starch is one of the major carbohydrates in the biosphere and the main source of energy in green plants. It is found as granules in grains, tubers, roots, leaves, fruits, and stems. Starch is composed of two main macromolecules, amylose and amylopectin. Starch granules have a wide range of sizes, categorized as large granule: 30–100 µm (e.g., potato, canna); medium granule: 5–30 µm (e.g., tapioca, barley) [1,2]; small granule: 2–10 µm (e.g., rice, oat); and lastly extremely small granule: 0.3–2 µm (e.g., quinoa and amaranth) [2,3]. Starch has gained attention from academic researchers and the industry as a food-grade ingredient due to multiple functionalities including the ability to act as emulsifiers in Pickering emulsions [4,5] as well as in the pharmaceutical area for drug delivery and encapsulation [6,7,8].

In order to improve the functionality of starch, starch in its native form can undergo modification. Such improvement techniques include chemical (esterification, oxidation, and etherification), physical (heat, milling), enzymatic, and genetic modifications. Chemical modification of starch by esterification/acylation is most extensively investigated technique. Examples of starch esterification include: modification with octenyl succinic anhydride (OSA) [5,9,10], fatty acids [11], citric acid [12], and folic acid [13]. Starch acylation involves the replacements of the available hydroxyl groups (OH group) of the glucose in starch structure (C-2, C-3, and C-6) with ester groups of the desired compound [13]. The maximum degree of substitution (DS) value that can be achieved is therefore equal to 3 [14]. Thus, the number of substituted groups, expressed as the number of hydroxyl groups substituted per glucose unit, is an important parameter for the characterization of modified starch. The chemical and physical properties of modified starch are also dependent on the reaction conditions (i.e., pH, time of reaction, temperature, and moisture content), type, molecular weight, and distribution of substituent groups [15].

**Figure 1:**
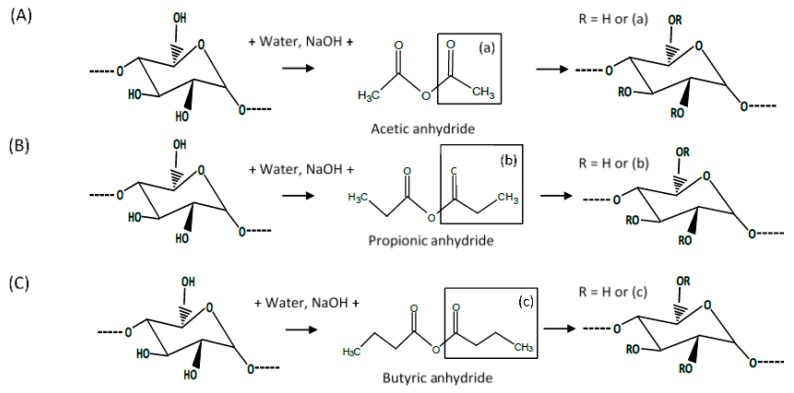
Schematic representation of the main reaction of starch acylation with short-chain fatty acid anhydrides (acetic, propionic, and butyric anhydride) (**A**) acetylation, (**B**) propionylation, and (**C**) butyrylation.

Esterification with fatty acid anhydrides, as shown in Figure 1, is a well-known chemical modification of starch that has been used for long-chain fatty acids [16], medium-chain fatty acids [17,18], and short-chain fatty acids [11]. Fatty acids chains are categorized into four groups, short (2–4 carbons), medium (6–12 carbons), long (14–18 carbons), and very long (with 22 carbons or more carbons) [19]. Acylated starch with short-chain fatty acids (SCFA starch) can be a useful chemical modification with improved technological and nutritional values. A study has proved that acylated starch, with DS values in the range of 0.20–0.25 had significant effects on short-chain fatty acid delivery to the large bowel [20].

Chemical modification by acylation is quantified by the determination of the degree of substitution (DS). Several analytical techniques have been introduced for DS determination such as titration-based methods (acidification and saponification) [21], Fourier-transform infrared spectroscopy (FTIR), headspace chromatography [22], and nuclear magnetic resonance (NMR) [16]. However, FTIR is normally employed to characterize the esterified products. To the date, only a few studies have been conducted to compare which methods are most efficient and reliable for DS determination. The most common standard method used is wet-chemistry also known as the titration-based method. This method is based on the hydrolysis of ester bonds in alkali solution and further titration to determine the percentage of acylation. This classic technique is considered straight forward and inexpensive, however this method is time consuming and the results are prone to be influenced by variable end-points during the titration. An alternative to titration is nuclear magnetic resonance, which is an advanced way to determine the DS value of starch as it provides quantitative chemical and structural information that could not be obtained by the titration method [23]. The position and DS of starch acylation were measured using NMR by the identification of protons and carbon shift of methyl groups in the anhydroglucose unit and anhydride groups [24]. The DS determination of starch by using NMR can be done through liquid-state NMR (^1^H-NMR, ^13^C-NMR, ^31^P-NMR, and ^17^O-NMR) and solid-state NMR (^13^C CP/MAS-NMR) [25,26]. FTIR measurements can be used to confirm esterified products by the determination of the absorbance intensity of acyl groups of samples based on the Beer–Lambert law [27]. The sample preparation for FTIR quantification of the DS is relatively easy and performed in dry conditions without the addition of solvents. In addition to the characterization of molecular properties, FTIR can also be used for DS determination. An alternative way of determining DS is by a stoichiometric calculation, which is discussed thoroughly in this present work.

Thus, the purpose of this study was to compare different techniques in determining the DS of acylated starches modified at different fatty acid alkyl chain lengths (i.e., by acetate, propionate, and butyrate) and concentrations. The determination of DS is crucial as a primary stage to validate the effectiveness of starch modification. In this respect, the development and comparison of efficient and reliable DS determination techniques would be highly appreciated by researchers in seeking the precise and accurate DS quantification.

## 2. Materials and Methods

### 2.1. Reagents

Commercial rice starch from *Oryza sativa* was obtained from Hermann Kröner GmbH, Ibbenbüren, Germany. Starch from *Chenopodium quinoa* was isolated according to a previous procedure [3]. Starches were then esterified with acetic anhydride (ROTIPURAN ≥99% p.a. ACS, ISO, CAS no: 108-24-7) and propionic anhydride (≥98.5% for synthesis, CAS no: 123-62-6) both from Carl Roth GmbH & Co. KG ( Karlsruhe, Germany) and butyric anhydride (>97.0%, CAS no: 106-31-0) from Merck KGaA (Darmstadt, Germany). Acetone (Rotipuran ≥99.8% p.a.) was purchased from Carl Roth GmbH & Co. KG (Karlsruhe, Germany). Sodium hydroxide solutions were prepared by dissolving NaOH pellets (p.a. >99%, Merck, Darmstadt, Germany) in distilled water and titration with exactly 0.05 M H_2_SO_4_ in order to establish approximately 0.7 M of NaOH. Potassium bromide for IR spectroscopy Uvasol^®^ was purchased from Merck (Darmstadt, Germany). Deuterated dimethyl sulfoxide d-6 (DMSO-d_6_) (Cas no: P2206-27-1) was purchased from Sigma Aldrich (St. Louis, MO, USA).

### 2.2. Acetylation, Propionylation, and Butyrylation of Quinoa and Rice Starches

Starch (70.0 g) was dispersed in 280 g of distilled water for 20 min by stirring with a magnetic bar and plate at 500 rpm. The pH of the dispersion was adjusted to 6.5 by titration with 0.7 M NaOH. At room temperature, the anhydride was gradually added (within approx. 20–40 min) with a burette parallel with 0.7 M NaOH to maintain the pH value of the suspension constant at pH 8.5 ± 0.2. When the desired amount of anhydride was added, the suspension was continuously stirred to allow the acylation reaction to continue under the controlled pH. The pH remained constant after 10–60 min, indicating that the entire amount of added anhydride had reacted with starch or OH^(−)^ groups, respectively. After the completion of the reaction, the pH was again adjusted from about 5.8–6.3 by the addition of the 0.7 M NaOH solution to 6.5. The molar amount of NaOH was determined throughout the duration of the reaction by monitoring the mass of the consumed NaOH solution using the electronic balance (Table 1). Knowledge of the mass, density, and exact molar concentration allows the calculation of the molar consumption of NaOH.

The product was then isolated by centrifugation (10 min, 3500× *g*) and was washed three times. Two washes were done by re-suspending the product in 420 mL of distilled water (15 min) and centrifugation (10 min, 3500× *g*). The final washing step was done by re-suspending the product in 400 mL of acetone and centrifugation. The final product of SCFA starch was dried at room temperature and conditioned for two days until it reached a constant weight.

### 2.3. Determination of the Degree of Substitution of Esterified Starches

#### 2.3.1. Stoichiometric Calculation from the Molar Amount of Educts

During the reaction of starch with fatty acid anhydrides (acetic, propionic, or butyric anhydride) in an aqueous weak alkaline solution, a main and a side reaction occurs. The main reaction of acylation is the reaction of the alkaline activated starch St–O^(−)^ with the anhydride. Meanwhile, a side reaction is a reaction of the anhydride with OH^(−)^– ions. The acylation reaction is stated as below:

Main reaction:

$${\mathrm{St}–\mathrm{OH}+\mathrm{OH}}^{\left(-\right)}+{\left({\mathrm{CH}}_{3}\mathrm{CO}\right)}_{2}O\;\to {\;\mathrm{St}–\mathrm{OCOCH}}_{3}{+\mathrm{CH}}_{3}{\mathrm{COO}}^{\left(-\right)}{+\;H}_{2}O.$$

Side reaction:

$${\left({\mathrm{CH}}_{3}\mathrm{CO}\right)}_{2}{O+2\mathrm{OH}}^{\left(-\right)}\to {\;2\mathrm{CH}}_{3}{\mathrm{COO}}^{\left(-\right)}{+H}_{2}O.$$

St–OH = starch,
(CH_3_CO)_2_O = acetic anhydride,
St–OCOCH_3_ = starch acetate.

In the main reaction, one mole of OH^(−)^ is needed for one mole of acetic anhydride. On the other hand, for the side reaction, two moles of OH^(−)^ are needed for pH neutrality. Therefore, the ratio of the molar amount of OH^(−)^ to the molar amount of anhydride must be between 1 (only main reaction) and 2 (only side reaction). The molar amounts of anhydride and NaOH, which were determined stoichiometrically in this section, are presented in Table 2 and Table 3. These values allow the calculation of the DS of the esterified starches directly from the reaction parameters.

Data from Table 1 show the educts which were used for the acylation of quinoa and rice starches. Three sodium hydroxide solutions with slightly different molarities were used for the acylation reactions. The density of all NaOH solutions was 1.026 g/mL at 22 °C.

The DS and AC were calculated stoichiometrically from the derived equation by considering the molar amount of educts. From the molar amounts of anhydride (n (Anhyd.)) and sodium hydroxide (n (NaOH)) presented in Table 2 and Table 3, the DS of SCFA starches were calculated by the following Equation (1) and (2):

(1) n (Anhyd.–St.) = 2 × n (Anhyd.) − n (NaOH),

(2) $$\mathrm{DS}\;=\frac{n\;\left(\mathrm{Anhyd}.–\mathrm{St}.\right)}{n\;\left(\mathrm{Anhyd}.\mathrm{Glu}\right)},$$

n (Anhyd.–St.) = molar amount of anhydride which is chemically bonded to the starch, n (Anhyd.Glu) = molar amount of anhydroglucose.

In order to determine the number of moles of anhydroglucose, the following Equation (3) was used:

(3) $$n\;\left(\mathrm{Anhyd}.\mathrm{Glu}\right)\;=\frac{m\;\left(\mathrm{Anhyd}.\mathrm{Glu}\right)}{\;M\;\left(\mathrm{Anhyd}.\mathrm{Glu}\right)\;},$$

m (Anhyd.Glu) = mass of Anhyd.Glu, M (Anhyd.Glu) = molar mass of Anhyd.Glu = 162.1 g/mol, 70.0 g rice starch (88.7% dry matter) = 62.09 g dry matter (0.3830 mol Anhyd.Glu), 70.0 g quinoa starch (88.6% dry matter) = 62.02 g dry matter (0.3826 mol Anhyd.Glu).

From the DS value, the acyl content (AC) was calculated by the Equation (4) below:

(4) $$\mathrm{Acyl}\;\mathrm{content}\;=\frac{\mathrm{DS}\;\times \;100\%\times M\;\left(\mathrm{acyl}\;\mathrm{group}\right)\;}{\left(\mathrm{DS}\;\times \;\left(M\;\left(\mathrm{acyl}\;\mathrm{group}\right)\;-\;1\frac{g}{\mathrm{mol}}\right)\;+\;M\left(\mathrm{Anhyd}.\mathrm{Glu}\right)\right)\;},\;$$

Molar mass of acetyl, M (acetyl) = 43.04 g/mol; Molar mass of propionyl, M (propionyl) = 57.07 g/mol; Molar mass of butyryl, M (butyryl) = 71.10 g/mol; Molar mass of hydrogen = 1 g/mol.

Reaction efficiency (RE) of starch modification was calculated using the following Equation (5) based on the starch modification reaction:

(5) $$\mathrm{RE}\;\left(\%\right)\;=\frac{n\;\left(\mathrm{Anhyd}.–\mathrm{St}.\right)}{n\;\left(\mathrm{Anhyd}\right)}\times \;100.$$

#### 2.3.2. FTIR Spectroscopy

The acyl content of starch esters was determined by a Tensor 37 FTIR spectrometer (Bruker, Bremen, Germany). FTIR spectra of KBr pellets were recorded in the range of 4000–400 cm^−1^ at a resolution of 2 cm^−1^. Modified starch, 1.5 ± 0.1 mg, was mixed thoroughly with a mortar and pestle with 250 mg of KBr. One hundred and fifty milligrams of the mixture was first evacuated for 2 min and then compressed with a force of 115 kN (8700 bar) for 3 min. The resulting pellets with a diameter of 13 mm and a thickness of 0.50 mm were then used for measuring the spectra in the transmission mode. The transmittance spectra were baseline corrected and converted into absorbance spectra. KBr pellets were prepared in triplicate and were used for the determination of the mean value ± standard deviation of the integrals.

#### 2.3.3. Nuclear Magnetic Resonance (^1^H-NMR)

The chemical structure of starch and DS of modified starches were identified by ^1^H-NMR spectroscopy. Experiments were performed on an Agilent UNITY Inova (Agilent Technologies, Ltd., Santa Clara, CA, USA) operating at 500 MHz for ^1^H-NMR. Samples were prepared by following a procedure from [26] with slight modification. Starch samples were dissolved in DMSO-d_6_ at 85 °C with constant stirring until clear solutions were obtained. Then, 500 µL of clear solutions was transferred to 5 mm NMR tubes. Samples were analyzed at 45 °C, with 4.8 s relaxation delay and 128 scans. Due to the hygroscopic nature of both DMSO-d_6_ and starch, samples in triplicates were freshly prepared before the analysis. The ^1^H-NMR spectra were analyzed using MestReNova 14.0.1 software (Santiago de Compostela, Spain), and the DS of modified starch was calculated using the following Equation (6):

(6) $$\mathrm{DS}\;=\frac{\left(A\times 4\right)}{\left(3\times C\right)},$$

where A is integral of the methyl signals and C is the integral of the proton signals of the anyhydroglucose unit [28].

## 3. Results and Discussion

### 3.1. Determination of Degree of Substitution

#### 3.1.1. Stoichiometric Calculation from the Molar Amount of Educts

The AC and DS of SCFA starches calculated from stoichiometric equation are shown in Table 2 and Table 3. It was observed that increasing the amount of acyl anhydride increased the amount of acyl group bonding to the starch, thus increased the DS values. However, at the same amount of acyl anhydride added, the DS and percentage of acyl obtained for SCFA-rice starches were slightly higher compared to those for SCFA-quinoa starches. The same finding was reported by [29] that discovered low-amylose starches are able to undergo better substitution than high-amylose starches thus increasing the DS. The amylose contents of rice and quinoa starches used in this study were determined previously [3], which revealed that rice starch has low amylose content (4.43% ± 0.78) compared to quinoa starch (20.95% ± 0.45). Other than amylose and amylopectin content, the packing arrangement of these macromolecules in starch may also influence the efficiency of acylation [29]. In addition, the DS of acylated starch appeared to be dependent on the length of acyl anhydride e.g., acetate has the shortest carbon length (C2), followed by propionate (C3) and butyrate (C4) anhydride. Meanwhile, reaction efficiency were not dependent on type and level of modification as shown in Table 4.

#### 3.1.2. Fourier Transform Infrared Spectroscopy

**Figure 2:**
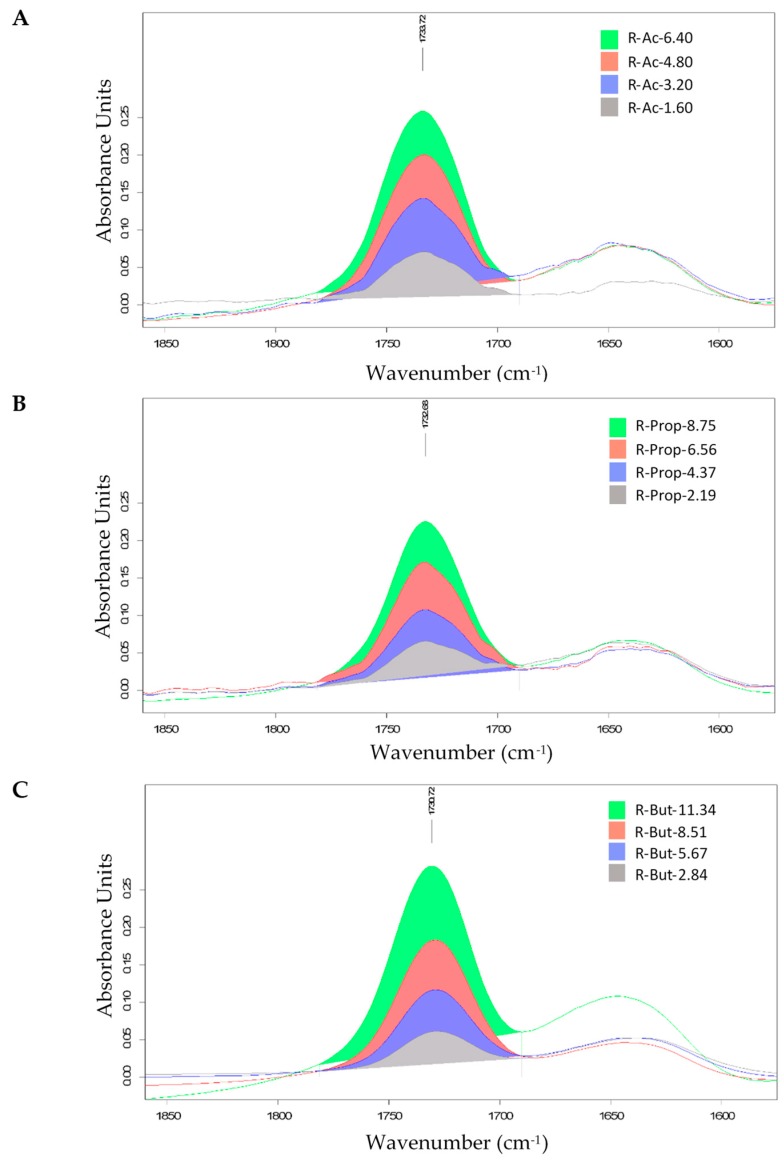
FTIR spectra with peak focused on carbonyl stretching vibration (C=O) band at integral (1781–1690 cm^−1^) of acetylated, propionylated and butyrylated rice starches at different levels of modification; (**A**) acetylated rice (R-Ac) (**B**) propionylated rice (R-Prop), and (**C**) butyrylated rice (R-But).

**Figure 3:**
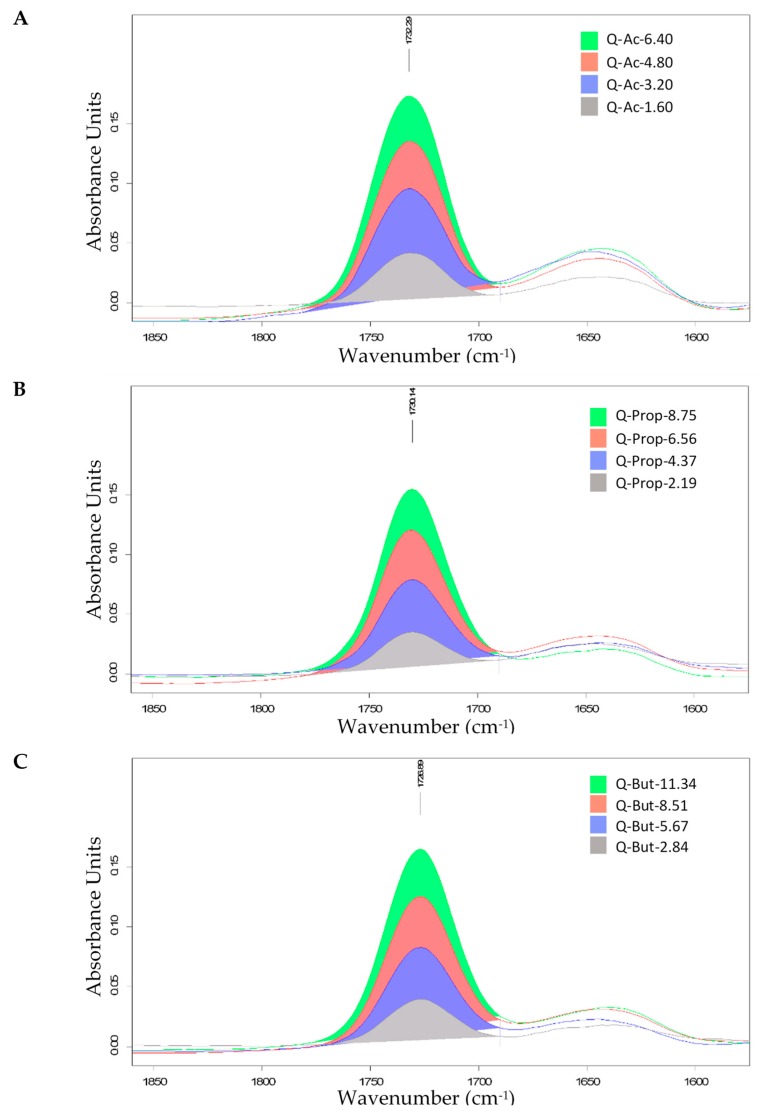
FTIR spectra with peak focused on carbonyl stretching vibration (C=O) band at integral (1781–1690 cm^−1^) acetylated, propionylated, and butyrylated quinoa starches at different levels of modification; (**A**) acetylated quinoa (Q-Ac), (**B**) propionylated quinoa (Q-Prop), and (**C**) butyrylated quinoa (Q-But).

A Fourier transform infrared spectroscopy study was conducted in order to determine and validate the conformational changes of SCFA starches compared to their native form. For the DS determination of SCFA starches by FTIR, the transmittance spectra were baseline corrected and converted into absorbance spectra. The absorbance spectra were normalized against the mass of the starch ester by setting the large absorption band of starch at 1020.3 cm^−1^ of all spectra to an absorbance of 2.000. By calculation of the differences between spectra (normalized modified starch vs.normalized native starch), a well-separated ester band was obtained. The carbonyl stretching vibration (C=O) band which ranged from 1781 to 1690 cm^−1^, with a maximum at approximately 1730 cm^−1^, was determined as shown in Figure 2 and Figure 3. By calibration of the integral of these bands with the DS values from the stoichiometric calculation in Section 2.3.1 (as a reference method), it is possible to obtain the DS of SCFA starches by FTIR sspectroscopy with a relatively low standard deviation.

**Figure 4:**
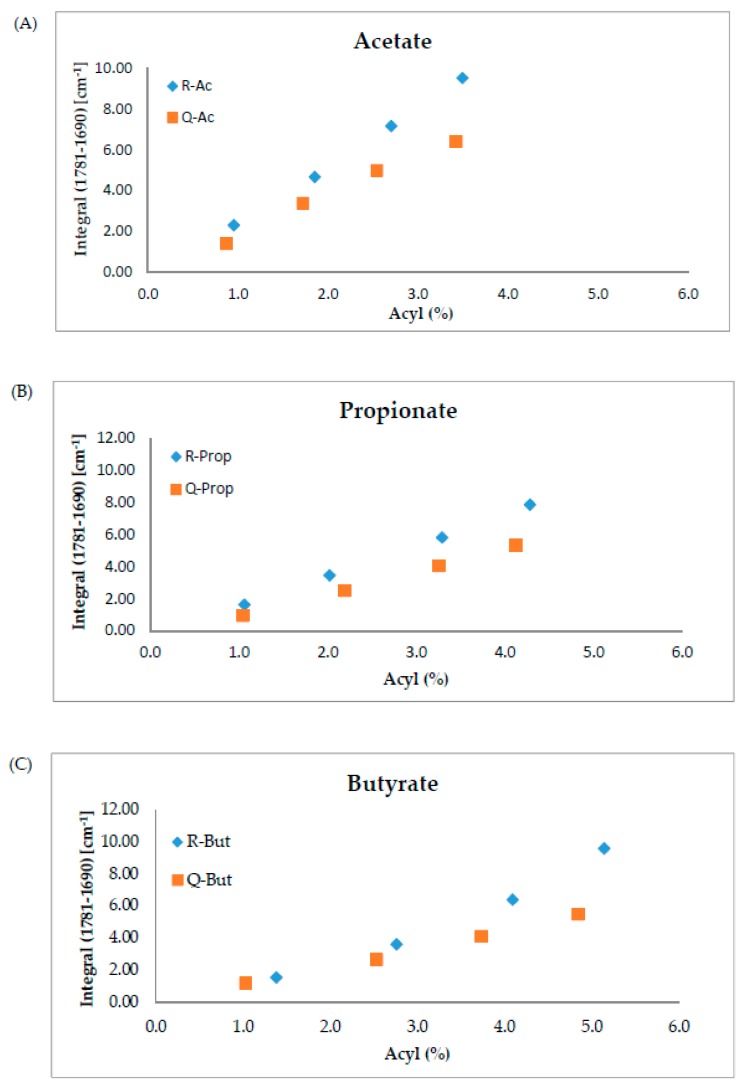
Correlation between acyl content obtained from stoichiometric equation and integral values of the carbonyl stretching vibration (C=O) band from FTIR of esterified starches; (**A**) acetylated rice and quinoa starches, (**B**) propionylated rice and quinoa starches, and (**C**) butyrylated rice and quinoa starches.

From the FTIR spectra depicted in Figure 2 and Figure 3, it is observed that SCFA starches containing the same acyl chain length showed a similar pattern with a peak at approximately 1730 cm^−1^. This shows that the acylation process has taken place and the starch was successfully esterified. The absorbance increased with the increase of DS or AC. In addition, a positive correlation was identified between integral calculated from the FTIR spectra and AC obtained by stoichiometric calculation as demonstrated in Figure 4 and Table 5.

#### 3.1.3. Nuclear Magnetic Resonance

**Figure 5:**
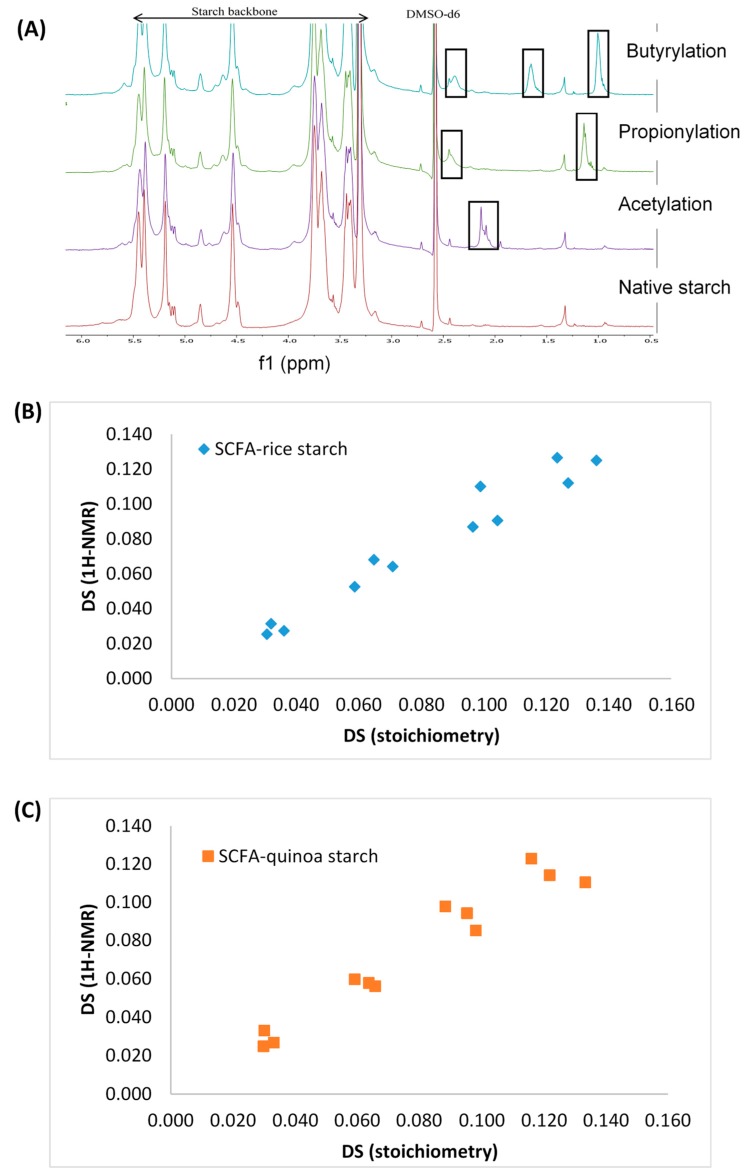
(**A**) Representative of 500 MHz ^1^H-NMR spectra of native starch and esterified short-chain fatty acid (SCFA) starches at different acylation processes, and correlation of DS obtained from ^1^H-NMR and stoichiometric calculation at a different level of modifications; (**B**) SCFA-rice starch; and (**C**) SCFA-quinoa starch.

Analysis of ^1^H-NMR was conducted as a reference method to determine the DS values of esterified starches. The resonance peaks observed in the ^1^H-NMR spectra were identified from their chemical shifts, and the four protons of the anhydroglucose part of the molecule have signals at 5.50 ppm (OH-3), 5.40 ppm (OH-2), 4.58 ppm (OH-6), 4.90 ppm (OH-4). The three methyl protons of the acyl chain of the acyl anhydride were assigned to the chemical shifts shown in the ^1^H-NMR spectra in Figure 5A. In this work, esterified starches show additional resonances, indicating the presence of acyl protons (methyl and methylene). The spectrum of acetylated starches showed a methyl group signal at 2.04 ppm, while the spectrum of propionylated starches shows two new peaks related to the methylene (2.33 ppm) and the methyl group (1.02 ppm). Butyrylation of starch gives rise to three new peaks observed at 2.27 and 1.55 ppm for the methylene groups and 0.89 ppm for the methyl group. As native starch does not contain an acyl group in the spectra, the presence of new peaks indicated a successful esterification process. In addition, as shown in FTIR spectra, the ^1^H-NMR spectra also showed the increasing intensity of assigned methyl and methylene peaks with increasing DS. The ^1^H-NMR analysis showed that the DS values obtained in this work were in close agreement with the DS obtained in the stoichiometric method as shown in Figure 5B,C, and Table 3.

Advanced techniques such as ^1^H-NMR can be used in DS determination, however, challenges in determining the appropriate solvents for the best H shift or minimizing impurities influence the DS determination by ^1^H-NMR [30]. For this reason, the use of the stoichiometric method in this work was verified as an alternative method to determine the DS values.

## 4. Conclusions

In this work, a new direct method for the quantification of DS values of SCFA starches has been developed by using stoichiometric calculations. The acylation and DS values obtained in this way were verified by FTIR and ^1^H-NMR and are in positive agreement with both methods. The stoichiometric calculation provides an efficient determination with no loss of sample and prevents sampling errors. An important aspect is that the DS is performed simultaneously during the acylation reaction on the entire amount of starch undergoing modification, thereby reducing error, as well as with the possibility for control of the reaction.

## Figures and Tables

**Table 1:** Educts for the reaction of short-chain fatty anhydrides with quinoa and rice starch.

Anhydride	Purity	Density (g/mL)	Molar Mass (g/mol)	Molarity of NaOH Used during Acylation (mol/L)	
				Quinoa Starch	Rice Starch
Acetic	≥99%	1.082	102.1	0.7095	0.7095
Propionic	≥98.5	1.015	130.1	0.7148	0.7170
Butyric	≥97.0%	0.967	158.2	0.7148	0.7170

**Table 2:** Reaction, product parameters and degree of substitution (DS) obtained from stoichiometry and ^1^H-NMR analysis of acetylated, propionylated, and butyrylated rice starch.

Volume Anhydride (mL)	Anhyd (mol)	NaOH (g)	NaOH (mol)	Anhyd.–St. (mol)	Acyl (%)	DS (stoichiometry)	DS (^1^H-NMR)
Ac-A 1.60	0.01679	28.63	0.01980	0.01378	0.95	0.0360	0.0274 ± 0.0012
Prop-A 2.19	0.01683	31.43	0.02196	0.01170	1.06	0.0305	0.0255 ± 0.0017
But-A 2.84	0.01684	30.74	0.02148	0.01220	1.38	0.0319	0.0314 ± 0.0025
Ac-A 3.20	0.03357	57.88	0.04002	0.02712	1.85	0.0708	0.0642 ± 0.0048
Prop-A 4.37	0.03358	64.03	0.04475	0.02241	2.02	0.0586	0.0527 ± 0.0026
But-A 5.67	0.03362	60.72	0.04243	0.02481	2.76	0.0648	0.0681 ± 0.0010
Ac-A 4.80	0.05036	87.84	0.06074	0.03998	2.70	0.1044	0.0906 ± 0.0030
Prop-A 6.56	0.05042	91.42	0.06389	0.03695	3.29	0.0965	0.0869 ± 0.0039
But-A 8.51	0.05046	91.19	0.06373	0.03718	4.09	0.0971	0.1102 ± 0.0029
Ac-A 6.40	0.06715	118.85	0.08219	0.05211	3.49	0.1361	0.1251 ± 0.0053
Prop-A 8.75	0.06724	122.88	0.08587	0.04865	4.28	0.1270	0.1121 ± 0.0007
But-A 11.34	0.06724	124.74	0.08717	0.04731	5.14	0.1235	0.1267 ± 0.0026

Abbreviations: Ac-A = acetic anhydride, Prop-A = propionic anhydride, But-A = butyric anhydride, n (Anhyd.) = mol of anhydride; n (Anhyd.–St.) = mole anhydride bound on starch.

**Table 3:** Reaction, product parameters and DS obtained from stoichiometry and ^1^H-NMR analysis of acetylated, propionylated, and butyrylated quinoa starch.

Volume Anhydride (mL)	Anhyd. (mol)	NaOH (g)	NaOH (mol)	Anhyd.–St. (mol)	Acyl (%)	DS (stoichiometry)	DS (^1^H-NMR)
Ac-A 1.60	0.01679	30.24	0.02091	0.01267	0.87	0.0331	0.0269 ± 0.0026
Prop-A 2.19	0.01683	31.94	0.02225	0.01141	1.04	0.0298	0.0250 ± 0.0015
But-A 2.84	0.01684	31.90	0.02222	0.01146	1.30	0.0300	0.0331 ± 0.0040
Ac-A 3.20	0.03357	60.72	0.04199	0.02515	1.72	0.0657	0.0562 ± 0.0020
Prop-A 4.37	0.03358	61.40	0.04277	0.02439	2.19	0.0637	0.0579 ± 0.0019
But-A 5.67	0.03362	64.03	0.04461	0.02263	2.53	0.0591	0.0598 ± 0.0053
Ac-A 4.80	0.05036	91.35	0.06317	0.03755	2.54	0.0981	0.0852 ± 0.0019
Prop-A 6.56	0.05042	92.38	0.06436	0.03648	3.25	0.0953	0.0943 ± 0.0002
But-A 8.51	0.05046	96.35	0.06713	0.03379	3.73	0.0883	0.0978 ± 0.0025
Ac-A 6.40	0.06715	120.45	0.08330	0.05100	3.42	0.1333	0.1105 ± 0.0058
Prop-A 8.75	0.06724	126.06	0.08783	0.04665	4.12	0.1219	0.1143 ± 0.0061
But-A 11.34	0.06724	129.36	0.09012	0.04436	4.84	0.1159	0.1229 ± 0.0065

**Table 4:** Reaction efficiency of acetylated, propionylated, and butyrylated rice and quinoa starch based on stoichiometry.

Volume Anhydride (mL)	Reaction Efficiency (%)	
	Rice Starch	Quinoa Starch
Native	not defined	not defined
Ac-A 1.60	82.1	75.5
Prop-A 2.19	69.5	67.7
But-A 2.84	72.5	68.1
Ac-A 3.20	80.8	74.9
Prop-A 4.37	66.7	72.6
But-A 5.67	73.8	67.3
Ac-A 4.80	79.4	74.6
Prop-A 6.56	73.3	72.4
But-A 8.51	74.6	67.0
Ac-A 6.40	77.6	76.0
Prop-A 8.75	72.4	69.4
But-A 11.34	70.4	66.0

**Table 5:** Acyl content obtained from stoichiometric equation and integral of the carbonyl stretching vibration (C=O) band of rice and quinoa starch esters in the range of (1781–690 cm^−1^).

**Rice Starch**					
**Acetate**		**Propionate**		**Butyrate**	
Acyl (%)	Integral (1781–1690) (cm^−1^)	Acyl (%)	Integral (1781–1690) (cm^−1^)	Acyl (%)	Integral (1781–1690) (cm^−1^)
0.95	2.31 ± 0.17	1.06	1.65 ± 0.14	1.38	1.55 ± 0.09
1.85	4.67 ± 0.62	2.02	3.47 ± 0.07	2.76	3.63 ± 0.11
2.70	7.16 ± 0.23	3.29	5.84 ± 0.17	4.09	6.41 ± 0.41
3.49	9.52 ± 0.38	4.28	7.89 ± 0.65	5.14	9.61 ± 0.45
**Quinoa Starch**					
**Acetate**		**Propionate**		**Butyrate**	
Acyl (%)	Integral (1781–1690) (cm^−1^)	Acyl (%)	Integral (1781–1690) (cm^−1^)	Acyl (%)	Integral (1781–1690) (cm^−1^)
0.87	1.41 ± 0.12	1.04	0.96 ± 0.06	1.03	1.19 ± 0.09
1.72	3.39 ± 0.32	2.19	2.51 ± 0.05	2.53	2.66 ± 0.03
2.54	5.00 ± 0.52	3.25	4.07 ± 0.15	3.73	4.12 ± 0.09
3.42	6.41 ± 0.08	4.12	5.34 ± 0.06	4.84	5.51 ± 0.10
